# Health Hints for Travelers

**Published:** 1884-08

**Authors:** 


**Health Hints for Travelers.** By JOHN C. SUNDBERG, M. D. D. G. Brinton, 115 South Seventh Street, Philadelphia. 1884.

It is bad enough to fall sick at home, but how much worse when among strangers. The object of this little book is a commendable one, and it may exert some influence, by the practical nature of its suggestions, in teaching the traveler to keep himself well.

